# Red Blood Cell Membrane Bioengineered Zr-89 Labelled Hollow Mesoporous Silica Nanosphere for Overcoming Phagocytosis

**DOI:** 10.1038/s41598-019-43969-y

**Published:** 2019-05-15

**Authors:** Jun Young Lee, Chirag K. Vyas, Gun Gyun Kim, Pyeong Seok Choi, Min Goo Hur, Seung Dae Yang, Young Bae Kong, Eun Je Lee, Jeong Hoon Park

**Affiliations:** Korea Atomic Energy Research Institute, Radiation Instrumentation Division, Jeongeup-si, 56212 Republic of Korea

**Keywords:** Membrane proteins, Positron-emission tomography, Cancer imaging, Nanoparticles

## Abstract

Biomimetic nanoparticles (NPs) have been actively studied for their biological compatibility due to its distinguished abilities viz. long-term circulation, low toxicity, ease for surface modification, and its ability to avoid phagocytosis of NPs by macrophages. Coating the NPs with a variety of cell membranes bearing the immune control proteins increases drug efficacy while complementing the intrinsic advantages of the NPs. In this study, efforts were made to introduce oxophilic radiometal ^89^Zr with hollow mesoporous silica nanospheres (HMSNs) having abundant silanol groups and were bioengineered with red blood cell membrane (Rm) having cluster of differentiation 47 (CD47) protein to evaluate its long-term *in vivo* behavior. We were successful in demonstrating the increased *in vivo* stability of synthesized Rm-camouflaged, ^89^Zr-labelled HMSNs with the markedly reduced ^89^Zr release. Rm camouflaged ^89^Zr-HMSNs effectively accumulated in the tumor by avoiding phagocytosis of macrophages. In addition, re-injecting the Rm isolated using the blood of the same animal helped to overcome the immune barrier. This novel strategy can be applied extensively to identify the long-term *in vivo* behavior of nano-drugs while enhancing their biocompatibility.

## Introduction

The morphological properties of nanoparticles (NPs), including their functionality, plays the crucial role in their biological applications^1–4^. Numerous previous studies have been attempted in the past to maximize the stability, circulation time, drug delivery efficiency and toxicity of NPs in biological system^5–7^. Polyethylene glycol-modified NPs (PNPs) have been studied as a ‘gold standard’ to perform these functions^8–10^. However, long-term circulation was one of the major hurdles and was not achieved in this case, due to the dense PNP corona^11^. While in contrast, cell membrane-coated (i.e. biomimetic) NPs are increasingly being used as drug delivery agents, because of their prolonged circulation time and effective tumor targeting properties. Surpassing the immune barrier using biomimetic NPs provides a new strategy for their application in biological studies. Cell membranes extracted from red blood cells (RBCs), white blood cells, platelets, bacteria, cancer cells and stem cells have been used in the previous studies^12–14^. In particular, RBCs have a lifespan of ~120 days before their immune-suppression by the body^15,16^. Phagocytosis of NPs uniformly coated by RBC membrane (Rm) is reduced by the expressed cluster of differentiation 47 (CD47) on the membrane^17–21^. The binding of CD47 to signal-regulatory protein-α (SIRP-α) induces the phosphorylation of the cytoplasmic tail of SIRP-α, which leads to the binding and activation of protein phosphatase, as well as the blocking of phagocytosis through inhibiting the motor protein myosin IIA accumulation in the phagocytic synapses. The Rm coated NPs (Rm-NPs) can inhibit the phagocytosis of macrophages by the corresponding pathway.

This approach of coating the NPs with the biomimetic membranes demonstrates an innovative drug delivery system for tumor diagnosis with a minimal dose of drugs. The prolonged blood circulation of Rm-NPs increases the enhanced permeability and retention (EPR) effect while reducing systemic toxicity with slow clearance. Hollow mesoporous silica nanospheres (HMSNs) are a highly desirable type of silica NP (SNP) in biomedical fields because of their versatility. Tuning the functional groups on HMSN surfaces widens the range of their applications. Furthermore, the hollow spaces in HMSNs can be loaded with specific drugs to treat a variety of diseases, including cancers^22–27^. In addition, HMSNs have abundant silanol groups (Si-OH) on their surfaces^28,29^ facilitating the introduction of various oxophilic radiometals, including ^89^Zr, ^68^Ga, ^111^In, ^90^Y, ^177^Lu and ^64^Cu^30^.

**Figure 1:**
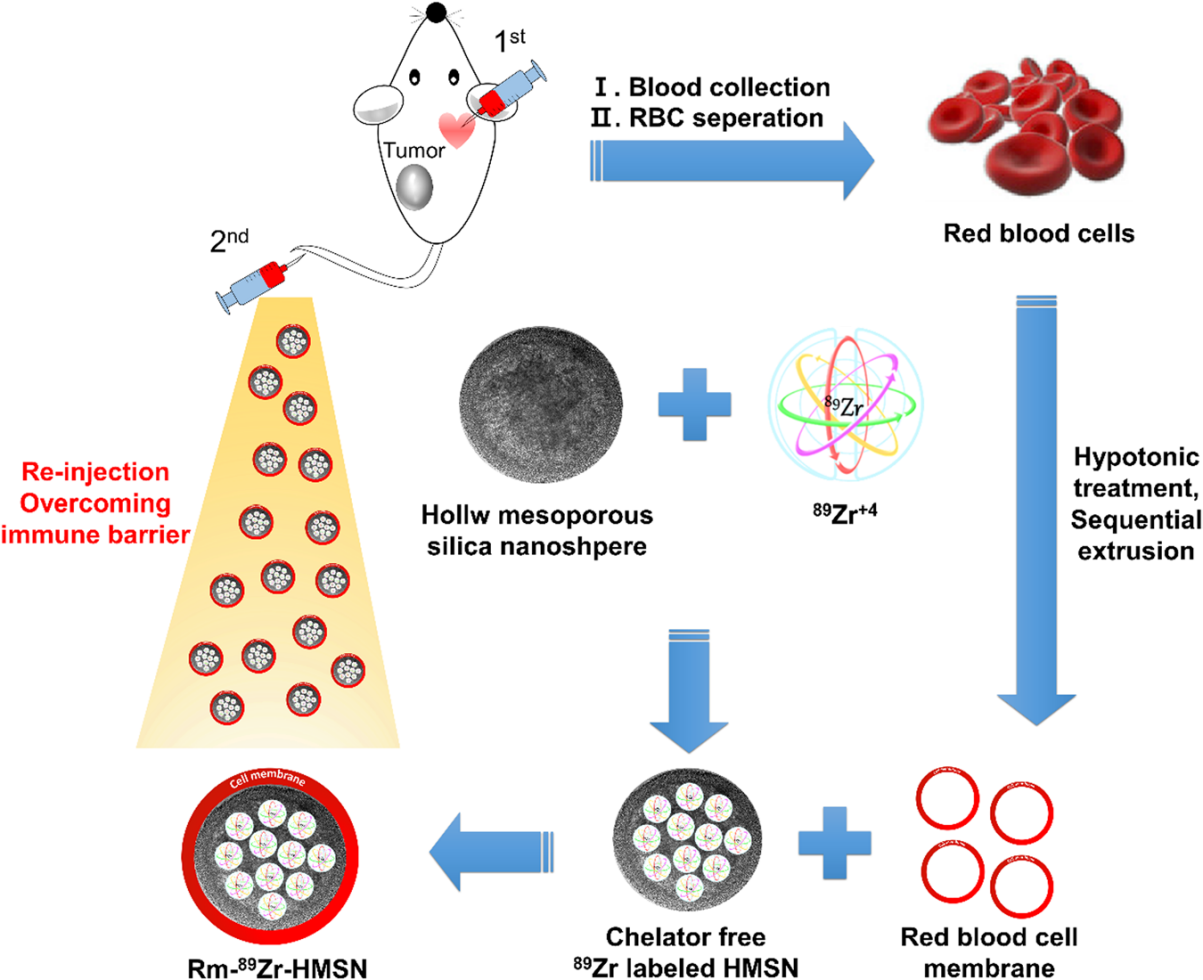
Stepwise representation of the synthesis of Rm-camouflaged ^89^Zr-labelled HMSNs. First, blood collection by <10% circulating blood volume from the mouse and second, re-injection for overcoming the immune barrier.

Non-invasive molecular imaging techniques like positron emission tomography (PET) fused with computed tomography/magnetic resonance imaging have undergone great development since their establishment and are set to play a consequential role in personalized medicine while offering utilitarian solutions for several diseases including cancers^31,32^. There has been a growing interest in the use of emerging radioisotopes (RIs) including but not limited to ^89^Zr (half-life; t_1/2_ = 78.4 h), ^64^Cu (t_1/2_ = 12.7 h), ^86^Y (t_1/2_ = 14.7 h) and ^68^Ga (t_1/2_ = 67.63 m) for PET^33^. These RIs have been studied extensively, and tagged to probes, including NPs, as carrier materials^34^. ^89^Zr displaying ease of onsite production, favorable nuclear decay properties and suitable conjugation chemistry is a promising long-lived RI for long-term biological distribution and *in vivo* tumor targeting studies^35^. Herein, we report for the first time the synthesis of Rm-coated, ^89^Zr-incorporated HMSNs (Rm-^89^Zr-HMSNs; Fig. 1) with enhanced blood circulation capabilities and examine their applications in drug delivery and/or as an imaging agent for PET. HMSNs were synthesized by the modified re-deposition method^36^. Reduced biodegradability and toxicity and enhanced long-term circulation were achieved by coating the HMSNs with Rm. In addition, long-term monitoring of Rm-HMSNs was enabled by incorporating the long-lived ^89^Zr on the silanol groups of the HMSNs.

## Results and Discussion

### Characterization and identification

Characterization and identification of nanoparticles were carried out using spectrometric analysis and dynamic light scattering system.

**Figure 2:**
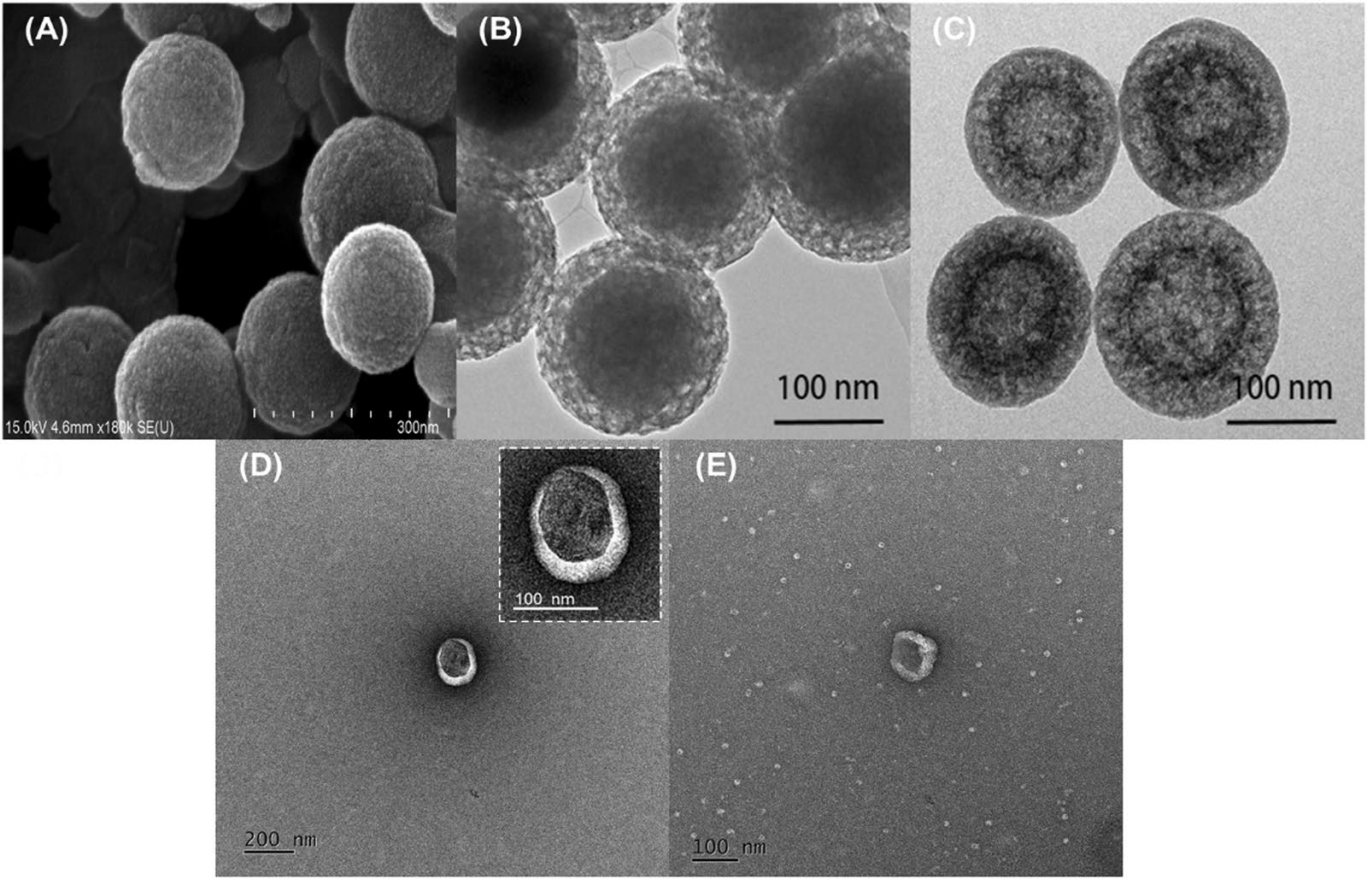
Representative SEM (**A**) and TEM (**B**–**E**) images of biomimetic nanoparticles; (**A**) HMSNs (**B**) Mesoporous silica nanoparticles coated SNP (**C**) HMSNs (**D**) Rm-HMSNs, and (**E**) Rm ghost with differently scale bar.

**Figure 3:**
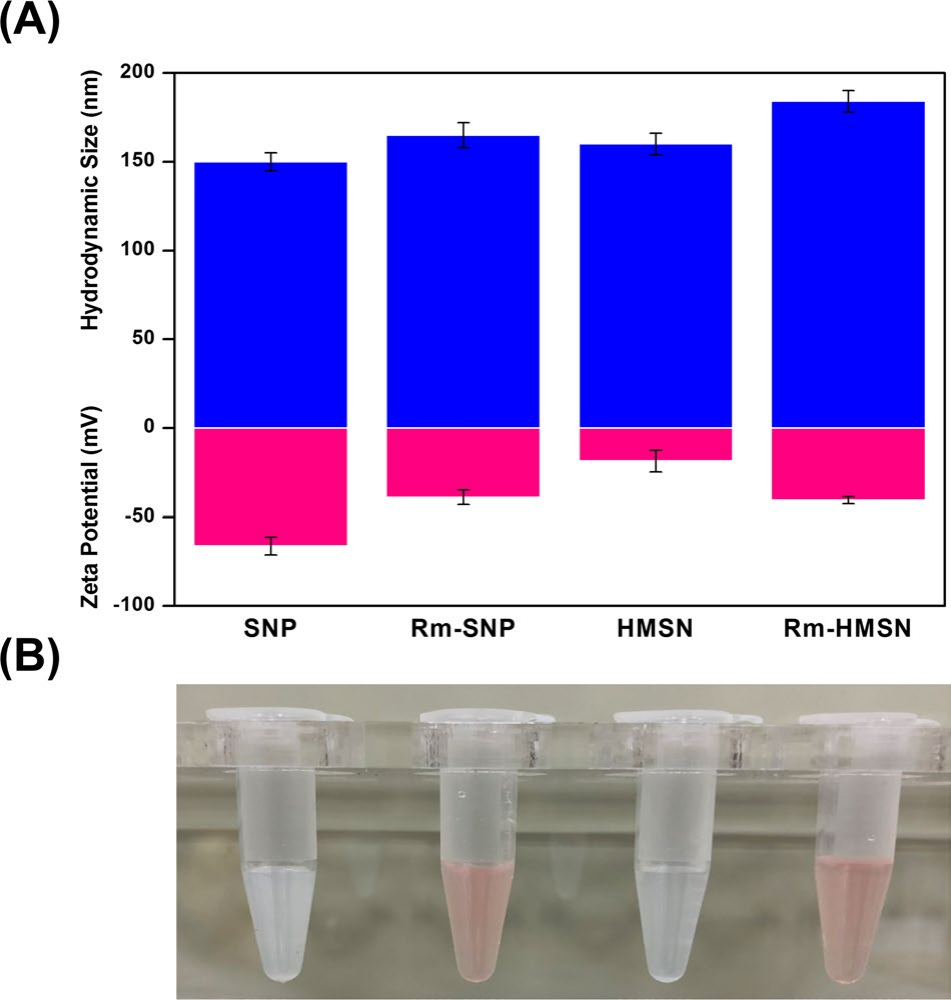
Physicochemical characterization. (**A**) Hydrodynamic size (top: blue bar) and Zeta Potential (bottom: pink bar) of SNP, Rm-SNP, HMSN, and Rm-HMSN (n = 3; mean ± s.d.). (**B**) The photographs of dispersion of the SNP, Rm-SNP, HMSN, and Rm-HMSN (500 µL, 1.0 mg/mL).

**Figure 4:**
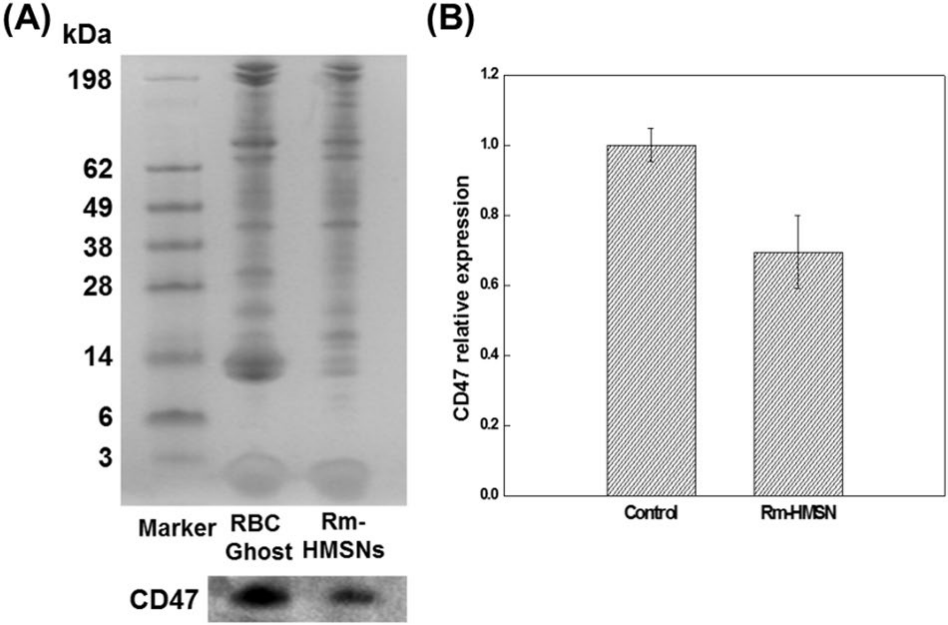
Proteins characterization of relative RBC membrane. (**A**) SDS-PAGE and Western blotting protein analysis of RBC ghost and Rm-HMSN (**B**) Expression levels of CD47 protein on RBC Ghost and Rm-HMSN.

IR absorption peaks of synthesized HMSNs showed common spectral bands at 801 ν_sym_ cm^−1^ (Si-O-Si), 965 ν_as_ cm^−1^ (Si-O), and 1106 ν_as_ cm^−1^ (Si-OH). Bands at 2854 ν_sym_ and 2924 ν_as_ cm^−1^ were assigned to the –CH_2_ group present in the intact CTAB, while a broad band at 3466 cm^−1^ was due to the water molecules adsorbed by the HMSN silanol groups (see Supplementary Fig. S1). The X-ray diffraction pattern indicates that the HMSN mesopores are disordered which is a typical pattern of mesopore NPs (see Supplementary Fig. S2). The thermogravimetric analysis (TGA) was carried out under static air up to 1070 °K at a heating rate of 10 °C/min. TGA of HMSN shows the weight loss due to dehydration and removal of free cationic surfactant (CTAB) at 150–300 °C with significant weight loss (~20%). It’s stability at higher temperature was confirmed by further increasing the temperature up to ~550 °C without any remarkable weight reduction (≤1%) (Supplementary Fig. S3). Scanning electron microscopy (SEM), and transmission electron microscopy (TEM) images (Fig. 2 and Supplementary Fig. S4) demonstrate HMSNs with a particle size of ~150 nm which can be loaded by various drugs into their mesopores, enhancing the EPR effect. The thickness of the HMSNs with coated Rm was increased by about ~20 nm, which has no significant change in particle size while maintaining its effective passive targeting property. SNPs and HMSNs with hydrodynamic sizes of 150 and 160 nm possessed zeta potentials of −66.2 and −18.6 mV (Fig. 3A) respectively. The negative charge was due to the presence of silanol groups on the NPs. HMSNs had a higher surface charge than SNPs with intact CTAB. Therefore, the oxophilic radiometals can be introduced easily and effectively into the HMSNs. The Rm-coated NPs (Rm-SNPs and Rm-HMSNs) had similar surface charges (−39.0 and −40.6 mV) due to the presence of sialylated glycoproteins on the Rm, preventing its agglomeration and enhancing its dispersion and diffusion abilities (Fig. 3B). Surface proteins of the Rm and Rm-HMSNs were identified by the sodium dodecyl sulfate-polyacrylamide gel electrophoresis (SDS-PAGE) and western blot. Protein bands of Rm and Rm-HMSNs were identical (Fig. 4A and Supplementary Fig. S5) suggesting the precise topological orientation of the Rm on the core of HMSNs which is crucial characteristics while performing their desired functions in this study. The level of CD47 expressed on the Rm-HMSNs was higher (RBC ghost 99.7% ± 4.70 and Rm-HMSNs 69.5% ± 10.4) (Fig. 4B). Lowered density of CD47 expressed by Rm-HMSNs is the direct consequence of losses occurred while its extrusion. The above mentioned results indicates successful translocation of the Rm on the surface of HMSNs and shows that the function of RBCs can be maintained and HMSNs can overcome immune-barrier.

### Cell viability

3-(4,5-dimethylthiazol-2-yl)-2,5-diphenyltetrazolium bromide (MTT) assay conducted with various concentrations of HMSNs and Rm-HMSNs (0–50 µg/mL) for 48 h showed low toxicity in both cases (Supplementary Fig. S6). However, at higher concentrations, the Rm-HMSNs displayed better results towards the cell viability as compared with bare HMSNs. The low toxicity of the Rm-HMSNs indicates that they could reduce systemic toxicity while displaying increased long-term circulation. It is important to note that Rm used to coat the NPs in this study were isolated using the blood samples collected from the heart of the same mouse later used to assess the *in vivo* behavior of NPs. Studies involving the radiotracer needs µg amount of NPs which will indeed require small amount of Rm, this was achieved by withdrawing <10% circulating blood volume from the mouse. This helped to overcome the immune barrier and toxicity involved in using Rm from the different sources.

### Radio-labelling and stability

We used ^89^Zr as a radio-tag to investigate the long-term biological behavior of Rm-HMSNs. ^89^Zr is osteophilic in nature and accumulates in the bone if not complexed with the suitable agent, enhancing its passive cumulation generating pseudo-images. However, using a bulky ligand with large surface area increases its interaction with several proteins, inhibiting its main function. Here, ^89^Zr was directly introduced into the SNPs and HMSNs through its interaction with the Si-O- group from silanol without a chelator. As a proof of concept, we examined the comparative labeling efficiency and stability of HMSNs labelled with ^89^Zr, ^18^F (the gold standard for PET) and ^68^Ga (an oxophilic radiometal) (Supplementary Fig. S9 and Table. S1). Based on bond dissociation energy, the oxophilicity trend was Zr > Ga > F which can be explained by the chelator-free binding of ^89^Zr and ^68^Ga to the silanol group^37^. The labeling efficiency and stability of ^18^F-HMSNs and ^68^Ga-HMSNs at 30 min were 2.93 ± 0.12% and 99.8 ± 1.68% and 28.7 ± 1.40% and 85 ± 1.22%, respectively. Although ^18^F and ^68^Ga are excellent positron emitters, their short half-life impedes their use as radio-tags to evaluate the long-term *in vivo* behavior of HMSNs.

The labeling efficiency of ^89^Zr towards HMSNs was ≥81.21% within the first 30 min and continued to increase to ≥96.14% at 24 h post-incubation. SNPs showed similar labeling efficiency (95.99% at 24 h) using ^89^Zr-chloride. As expected, ^89^Zr-labeling efficiency was dependent on the reaction time, where longer reaction time produced higher labeling yields (Supplementary Fig. S7). The stability studies with ^89^Zr-HMSNs and Rm-^89^Zr-HMSNs confirmed that ^89^Zr incorporated HMSN with Rm was quite stable as compared with uncoated HMSNs. Rm coated HMSNs while avoiding the release of Zr-89 may provide with the tool for long term *in vivo* studies of the system. (Supplementary Table S2)

### *In vitro*; cancer cell internalization

Cellular uptake of Rm-^89^Zr-HMSNs was high and fast within 1 hour (73.93 ± 3.04%), but was rapidly released from surface of cancer cell membrane because of weak surface affinity of cancer cells towards Rm-^89^Zr-HMSNs. The internalization of Rm-^89^Zr-HMSNs tends to increase over 24 hours (33.73 ± 1.42%), followed by an internalization of Rm-^89^Zr-HMSNs gradually. Effective internalization rate of Rm-^89^Zr-HMSNs appears higher as compared to that with the previously reported studies^38,39^. Based on these results, Rm-^89^Zr-HMSNs can reach the tumor site effectively during circulation in the blood. (Supplementary Fig. S8).

### *Ex vivo*; biodistribution

**Figure 5:**
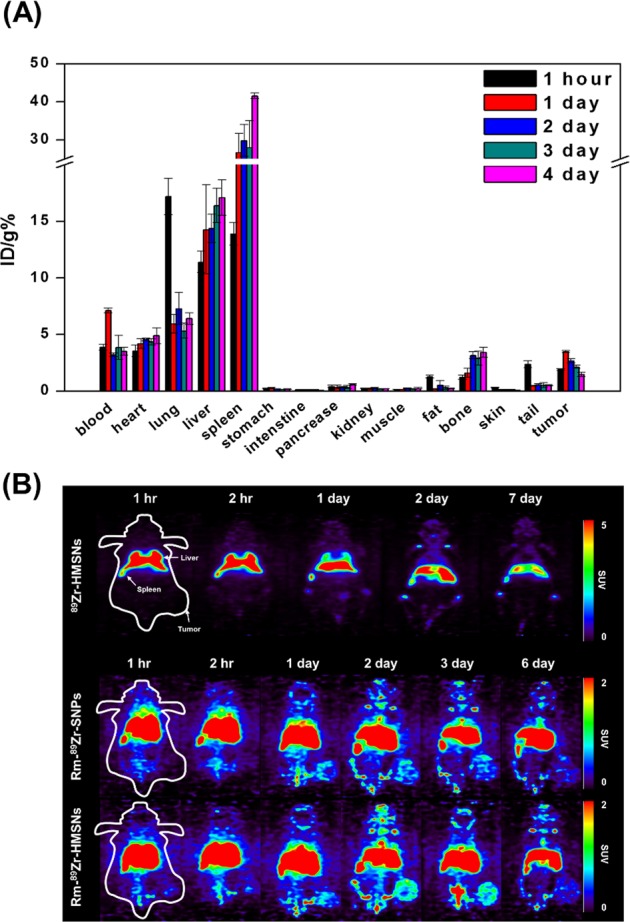
(**A**) Biodistribution study of Rm-^89^Zr-HMSNs in CT-26 tumor-bearing female Balb/c mice (n = 3 per group). (**B**) Small animal PET images of CT-26 tumor-bearing female Balb/c mice with ^89^Zr-HMSNs (top), Rm-^89^Zr-SNPs (middle) and Rm-^89^Zr-HMSNs (bottom). PET images were obtained for 1 week after tail intravenous injection of ^89^Zr labelled NPs.

*Ex vivo* biodistribution studies with Rm-^89^Zr-HMSNs using CT-26 tumor xenograft-bearing Balb/c mice after intravenous (i.v.) injection with the time interval of 96 h are shown in Fig. 5A. Although, accumulation values of Rm-^89^Zr-HMSNs in the liver and spleen were approximately 17.09 ± 1.58 and 41.53 ± 0.75 ID/g%, respectively, it shows a significantly lower uptake than previous studies^40,41^. The low accumulation in the liver and spleen is accompanied with high uptake in the tumor. The maximum tumor uptake for Rm-^89^Zr-HMSNs was observed at 24 h, with 3.48 ± 0.09 ID/g% with 7.11 ± 0.22 ID/g% in the blood (see Supplementary Tables S3 and S4). To compare the relative uptake in tumors and organs, the Rm-^89^Zr-HMSN uptake ratios in tumors and blood and other internal organs were measured after i.v. injection. Tumor to organs/blood ratios were highest at 48 h suggesting the incorporation of RIs with the long half-life to evaluate the *in vivo* performance of the nano-drugs.

### *In vivo*; PET imaging of radio-labelled NPs

Small animal PET studies with ^18^F-HMSNs, ^68^Ga-HMSNs, ^89^Zr-HMSNs, Rm-^89^Zr-SNPs and Rm-^89^Zr-HMSNs (Fig. 5B and Supplementary Fig. S9) provide insights on their biological behaviors. Although the accumulation of both ^89^Zr-HMSNs and Rm-^89^Zr-HMSNs could be found in major organs. It should be perceived that, Rm-^89^Zr-HMSNs exhibited significantly lower accumulation in liver, lung and spleen than that of ^89^Zr-HMSNs. PET image pattern confirms that Rm-^89^Zr-HMSNs was evenly distributed in the whole body. It can be well predicted from the images that the Rm is remains intact on the surface of HMSNs for more than 7 days. In addition, as liver is core organ of reticuloendothelial system (RES) for removing exogenous invader, will be a major hurdle hampering the circulation of HMSNs. Rm coating could enable HMSNs to effectively escape RES recognition, improving the circulation time^42^. The root cause of Rm coated HMSNs behavior is that CD47 functions as an inhibitor of phagocytosis through interaction of SIRPα expressed on phagocytes, leading to tyrosine phosphatase activation and inhibition of myosin accumulation at the sub-membrane assembly site of the phagocytic synapse^43^. In this way, CD47 serves as a “don’t eat me signal” while the loss of CD47 leads to homeostatic phagocytosis of NPs. CD47^−/−^ RBCs were readily taken up into macrophages and nonprofessional phagocytes by processes similar to apoptotic cell removal^44^. As mentioned above, we found that a minimum of ~24 h was required for solid tumor penetration of Rm-^89^Zr-HMSNs, with a saturation point at ~48 h. For radioimmunoimaging, proteins need around 24–48 h to penetrate in a solid tumor^45–48^. The blood circulation time of RBCs being ~120 days, it is a good approach to coat HMSNs using Rm to achieve optimal tumor to background ratio. Another factor one needs to consider is the half-life of radionuclide supposed to be used for radioimmunoimaging. ^89^Zr with the long half-life of 3.3 d is predominantly suitable in combination with proteins which allows imaging at later time points as compared to that with the conventional radionuclides for gaining optimum information^49^. ^18^F and ^68^Ga-labelled HMSNs did not display significant PET signals at the tumor sites, suggesting no tumor uptake, which can be attributed to the long-term circulation time needed for Rm-HMSNs to travel to the tumor site combined with the short half-life of ^18^F and ^68^Ga. Thus, the application of Rm-coated ^18^F-HMSNs and ^68^Ga-HMSNs is illogical for the long-term studies of Rm-HMSNs. In addition, although the distribution of Rm-^89^Zr-SNPs was similar to Rm-^89^Zr-HMSNs, the tumor uptake was low even when the same amounts of biomimetic NPs were administered, indicating their lower ability to circulate in the blood because of low-fluidity of SNPs. Uptake of Rm-^89^Zr-HMSNs by tumors was clearly observed at 24 h, with good tumor-to-background contrast, while avoiding the clearance through phagocytosis. According to previous studies on the association of nano-medicines without stealthy strategies^50^, increased uptake by macrophages is affected by particle shape, size and surface modification. In order to suppress the internalization of NPs by the mononuclear phagocytic system, it has been widely used to introduce a PEG ligand on the surface of NPs. However, the PEGylated nanoparticles can significantly reduce the ligand-targeting bioactivity^51^. In this study, Rm was used as an alternative bio-material and Zr-89 was introduced without chelator to avoid any change in the functional properties of the Rm. Particularly, bare NPs can be accumulated with high uptake in the liver, lung and spleen, which can cause high toxicity during long-term circulation, it has a low circulation half-life, which cannot be evaluated for effective targeting. On the other hand, the whole-body distribution results show high blood circulation half-life and avoidance of phagocytosis.

### *In vivo*; blood clearance

**Figure 6:**
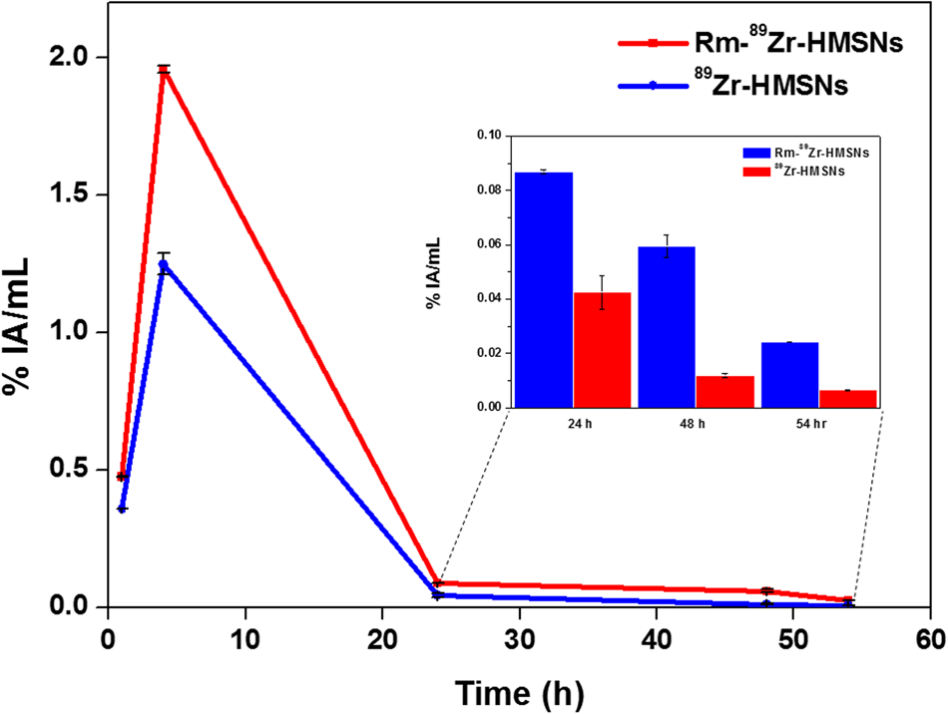
*In vivo*; Blood clearance curves were obtained for 54 hour after tail intravenous injection of ^89^Zr labelled NPs (n = 3; mean ± s.d.).

Blood clearance of ^89^Zr-labelled HMSNs and Rm-HMSNs was studied in healthy Balb/c mice. The blood retention of Rm-^89^Zr-HMSNs significantly was enhanced over a span of 54 h, compared to the ^89^Zr-HMSNs. As shown in Fig. 6, Rm-^89^Zr-HMSNs proved that circulation half-life of HMSNs can be prolonged. However, the HMSNs were rapidly cleared out of blood stream because of phagocytosis. The retention of ^89^Zr labelled HMSNs and Rm-HMSNs in the blood is 1.25% AI/mL and 1.96% Al/mL, respectively. These results indicate the successful translocation of Rm onto HMSNs surface suggesting that Rm-^89^Zr-HMSNs have the merit of long-term circulation.

## Conclusion

These studies execute successful synthesis of Rm-^89^Zr-HMSNs, having excellent fluidity, stability, and long-term circulation ability in biological systems. Since Rm-HMSNs are biomimetic NPs, they have low toxicity too. While having a promising behavior of chelator-free labeling of the ^89^Zr to the silanol group with high labeling efficiency (≥96%), Rm coated HMSNs further evades macrophage-mediated phagocytosis in the immune system and prevents release of ^89^Zr. Furthermore, this strategy validated that active targeting can not only be easily achieved and studied by introducing various cell membranes and target ligands but also the introduction of radionuclide into the biomimetic nano-platform can be used as a strategic tool for diagnosis and treatment of various diseases.

## Materials and Methods

### Materials

Tetraethyl orthosilicate (TEOS), Hexadecyltrimethylammonium bromide (CTAB), Sodium carbonate, 3-(4,5-dimethylthiazol-2-yl)-2,5-diphenyltetrazolium bromide (MTT), Oxalic acid, Diethylene triaminepentaacetic acid (DTPA), Ethylenediaminetetraacetic acid (EDTA), Sodium hydroxide, 4,7,13,16,21,24-Hexaoxa-1,10-diazabicyclo[8.8.8] (K2.2.2), Potassium carbonate, Human serum, HEPES buffer solution (1 M in H_2_O), Ethanol (ACS grade), Methanol (Sigma-Aldrich, HPLC grade), and Ammonium hydroxide (28~30% in water). Deferoxamine mesylate (USP, DFO), Phosphate buffered saline (Gibco, PBS, pH 7.4), Trifluoroacetic acid (DAEJUNG, TFA), BCA (Bicinchoninic acid) assay kit, Sodium dodecyl sulfate (SDS)-polyacrylamide gel, Lithium dodecyl sulfate (LDS) buffer, Enhanced chemi-luminescence (ECL) kit, Polyvinylidene difluoride (PVDF) membrane (Thermo-Fisher), Dulbecco’s modified eagle medium (Thermo-Fisher, DMEM), Fetal bovine serum (Thermo-Fisher, FBS), and Zr-resin (TRISKEM), Yttrium metal foil 0.64 mm ~99.5% (Alfa Aesar), Hydrochloric acid trace metal grade (Thermo-Fisher) were obtained and used without further purification. All animal experiments were progressed according to institutional animal care and use committee (IACUC) guidelines provided by Korea Atomic Energy Research Institute.

### Preparation of hollow mesoporous silica nanospehers

SNPs were synthesized by Stӧber method^52^. Briefly, 10 ml of ammonia solution and 6 ml of distilled water (D.W) were added in 250 ml of ethanol at 30 °C to which 40 ml of TEOS was added rapidly. Reactants were stirred constantly for two hours following which it was centrifuged and thoroughly washed with ethanol and water.

Mesoporous silica coated SNPs (SNP@mSNPs) were further synthesized using 0.15 g of CTAB and dissolved in 30 ml of water following which 30 ml of ethanol and ammonia solution were added. This solution was homogenised by stirring it for 30 min and 0.1 g of SNPs dispersed in 20 ml of distilled water was added. Next, 0.25 mL of TEOS was rapidly added and then stirred for 6 h. The reactant was centrifuged, washed and re-dispersed in 20 ml of water. Finally, HMSNs were synthesized by re-deposition method. The SNP@mSNPs dispersion was stirred for 12 h to which 0.46 g of sodium carbonate was added and stirred at 80 °C for 10 h after which it was suspended in mixture of methanol(16): HCl (1) solution and refluxed for 24 h at 80 °C. The product was centrifuged and washed by D.W.^53^.

### Characterization

NPs were recorded on a Perkin-Elmer FT-IR and prepared by classical technique KBr pellet. Removal of moisture and free CTAB from HMSNs were monitored thermogravimetrically (model 2950, TA Instruments, USA). Morphology and size of NPs were measured by scanning electron microscope (FE-SEM S-4200, Hitachi, Japan), transmission electron microscope (TEM, JEM 2011, Jeol, Japan) and bio-TEM (Tecnai G2 spirit Twin, FEI, USA). XRD pattern of the prepared HMSN powder was conducted using an X-ray diffractometer (Phillips X’pert MPD diffractometer, Almelo, Netherlands) with copper K-α target (40 kV, 30 mA). Data was collected at the 2-theta angle ranging from 3 to 90 with a scanning speed of 0.4 deg/min. To determine the hydrodynamic sizes and zeta potentials of SNPs, HMSN, Rm-SNPs and Rm-HMSN, as prepared suspensions were diluted (350 ppm) with D.W. The modified surface dependent hydrodynamic size and surface charge were determined using Zetasizer Nano ZS (Malvern instruments Ltd).

### Characterization of cell membrane proteins

CD47 proteins on the Rm-HMSNs were quantified using Western blotting analysis. Characterization of the Rm proteins, emptied RBCs and Rm-HMSN were treated with LDS lysis buffer. Then, the samples were denatured at 85 °C for 2 min and proteins of Rm and Rm-HMSN were quantitated using BCA assay kit. 25 µg of each protein was added into each well in a 4–12% sodium dodecyl sulfate-polyacrylamide gel. The gel was run at 200 V for 22 min and polyacrylamide gel was stained by coomassie brilliant blue according to the provided protocol before imaging. After separation of protein by SDS-PAGE, proteins transferred onto a PVDF membrane. After blocking the membranes with milk blocking buffer, the membrane was incubated using CD47 primary antibody (1:500) and IgG secondary antibody (1:1,000) was incubated for 3 h. Expressed CD47 protein onto Rm and Rm-HMSN was detected for the luminescence using an ECL kit by Chemi-doc (iBright FL-1000 Imager, Thermo-Fisher Scientific)

### Labelling efficiency of ^89^Zr-labelled SNP and HMSN

SNP and HMSN were dispersed in a HEPES buffer (pH 7.5; 0.1 M, 0.5 mL) at various concentrations (15, 50, 100, 200, 400 and 1,000 µg/mL). The pH of ^89^Zr-oxalate and ^89^Zr-chloride (~20 MBq) was adjusted to 7.5–8 using a Na_2_CO_3_ solution to which dispersed nanoparticles solution was added. After stirring at room-temperature for 24 hours, labelling efficiency was confirmed by radio thin-layer chromatography (radio-TLC) using 50 mM DTPA as the mobile phase^29^.

### Red blood cell membrane camouflaged ^89^Zr-HMSN and ^89^Zr-SNPs

RBCs were isolated using the blood of CT-26 bearing female Balb/c mouse withdrawn by cardiac puncture (<10% of circulating blood volume, re-use the blood drawn from same mice to avoid immune barrier). Mouse blood was collected into the 500 μL syringe (24 G) containing 8 μL of 0.5 M EDTA and was centrifuged (1500 rpm, 5 min, 4 °C) to separate the serum, buffy coat, white blood cell and platelet layers sequentially. Separated RBCs were washed with ice cold 1 × PBS. The purified RBCs were hemolyzed by 0.25 × PBS treatment for extraction of Rm then washed by centrifugation (13,500 rpm, 5 min) with ice 1 × PBS. The Rm was re-suspended in 1 × PBS and sonicated for 3 min at power of 130 W. After sonication the Rm fragments were extruded (Avanti, mini-extruder) sequentially through 400 nm and 200 nm polycarbonate (PC) membrane filters. The prepared Rm and ^89^Zr labelled NPs were mixed and extruded by 200 nm of PC membrane for at least 10 passes. Rm coated ^89^Zr-NPs were centrifuged and washed to remove uncoated Rm.

### Cell culture and animal tumor modelling

All cancer cell lines (CT-26, KB and A549) were cultured in DMEM and supplemented with 10% fetal bovine serum. Cell lines were maintained at 37 °C in a humidified atmosphere containing 5% CO_2_. CT-26 transplanted mice were prepared with female Balb/c mice. The cancer cell lines (5 × 10^6^ cells) were injected in the right thigh. The tumors were allowed to grow for 2–3 weeks before *ex vivo* and *in vivo* animal experiment.

### *In vitro*; cell viability

1 × 10^4^ cells/well were cultured in micro well plates and exposed to different weight of nanoparticles (0–50 µg/mL) and added 10 µL of MTT reagent to each well, including controls and incubated for 48 hr at 37 °C until a purple coloured formazan. It was dissolved in 100 µL of detergent to all wells. Then, supernatant was measured at 570 nm using a microplate reader model.

### *In vitro*; cellular uptake and internalization

The cellular uptake and internalization of Rm-^89^Zr-HMSNs was measured with CT-26, KB and A549 cells. The cancer cells were sub-cultured in 24 well plates (5 × 10^5^ cells/well) and incubated for 0.5, 1, 4, 24 and 48 hours. After incubation, the supernatant was removed and the cell pellets were washed by cold PBS to remove the unbound Rm-^89^Zr-HMSNs. Trypsin solution was used to harvest NPs from the internalized cancer cells. Following this, the cancer cell pellets were treated with 0.1 M sodium citrate for 5 min to remove surface bound Rm-^89^Zr-HMSNs. The radioactivity of unbounded Rm-^89^Zr-HMSNs and cells were measured by gamma counter and results were calculated as the accumulation ratio (%) ± standard deviation (s.d.).

### *Ex vivo*; biodistribution

Biodistribution studies were performed in compliance with the animal experimental guidelines and ethics approved by Korea Atomic Energy Research Institute (IACUC-2015-004). For biodistribution studies using the Rm-^89^Zr-HMSN, 1.85~2.59 MBq of Rm-^89^Zr-HMSN was injected by tail i.v in mice (n = 3 for each groups). Mice were dissected at defined time points: 1, 24, 48, 72 and 96 hours after i.v injection and the blood was collected from the heart and other organs viz. heart, lung, liver, spleen, stomach, intestine, pancreas, kidney, muscle, fat, bone, skin, tail, brain and tumor. The collected blood and organs were counted for accumulated Rm-^89^Zr-HMSN per organ weight relative to the injected Rm-^89^Zr-HMSN by measuring the radioactivity with a gamma counter. The results of biodistribution were expressed as the percentage of injected dose per gram (% ID/g).

### *In vivo*; small animal PET images

CT-26 bearing mice were used to identify pharmacokinetic pathways and tumor accumulation. The mice were anesthetized by exposing it to 1.5~2% isoflurane in oxygen. PET images were obtained at 1, 2, 24 and 48, 72 and 144 h of interval after intravenous (i.v) injection through tail vein in mice with of ^18^F-HMSN, ^68^Ga-HMSN, Rm-^89^Zr-SNPs, ^89^Zr-HMSN and Rm-^89^Zr-HMSN.

### *In vivo*; blood clearance

Once under anesthesia, Normal Balb/c mice (n = 3 per group) were injected intravenously via the tail vein with ~3.7 MBq ^89^Zr-HMSNs and Rm-^89^Zr-HMSNs. Blood samples (100 μL) were collected from the saphenous vein at a series of time points (1, 4, 24, 48 and 54 hour) into a γ-counting tube. Blood radioactivity was measured in a γ-counter and expressed as a percentage of the injected activity per mL (% IA/mL). The blood radioactivity at each time point was calculated including the decay correction.

## Supplementary information

Supplementary Dataset 1
