# Supplementary material for: Red Blood Cell Membrane Bioengineered Zr-89 Labelled Hollow Mesoporous Silica Nanosphere for Overcoming Phagocytosis
Source: Sci Rep. 2019 May 15;9:7419. doi: 10.1038/s41598-019-43969-y (PMC6520393; doi:10.1038/s41598-019-43969-y)
Supplement: Supplementary file 1 — Supplementary Dataset 1 [file 41598_2019_43969_MOESM1_ESM.pdf]

Red Blood Cell Membrane Bioengineered Zr-89 Labelled Hollow Mesoporous Silica Nanosphere for Overcoming Phagocytosis

*Jun Young Lee, Chirag K. Vyas, Gun Gyun Kim, Pyeong Seok Choi, Min Goo Hur, Seung Dae Yang, Young Bae Kong, Eun Je Lee, Jeong Hoon Park**

Jun Young Lee et al., Supplementary Method

Production of ^{89}Zr (Ref. 1)

^{89}Zr was produced by ^{89}Y(p, n)^{89}Zr reaction at RFT-30 an indigenous 30 MeV prototype cyclotron (Advanced Radiation Technology Institute, Korea Atomic Energy Research Institute) using the optimized irradiation conditions mounting the ^{89}Y metallic target on solid target irradiation station. In a nutshell, ^{89}Y (99.95%) was mounted at the irradiation station cooled with water jet. The initial 30 MeV of proton energy was degraded using 3.1 mm of aluminum degrader and the calculated incident proton energy on target was ~12.8 MeV. The target was irradiated using 12.8 MeV protons at 30 μA current for 2 hours producing 1.6 mCi/μA.h of ^{89}Zr with ≥ 99.99 % of radionuclidic purity. Irradiated target was allowed to cool for one hour and manually demounted and shifted to the hot-cell. The bulk target was dissolved in 6 M HCl (trace metal basis) after which hydroxamate based extraction chromatographic resin was employed for separation and purification of ^{89}Zr in oxalate and chloride form with ≥99.9% of radiochemical purity. The purified ^{89}Zr was employed for further labelling and bio-distribution studies.

Preparation of ^{18}F-HMSN and ^{68}Ga-HMSN

^{18}F was produced by ^{18}O(p, n)^{18}F reaction at RFT-30 an indigenous 30 MeV prototype cyclotron (Advanced Radiation Technology Institute, Korea Atomic Energy Research Institute). The produced ^{18}F was separated and solvent exchanged through a QMA cartridge with K2.2.2/K_2CO_3. 370 MBq of ^{18}F was stirred in acetonitrile with HMSN of 1 mg at 80 °C for 3 hours. After the addition of trifluoroacetic acid (20 μL), the reaction was allowed to proceed at 80 °C for 1 hour. To remove “free ^{18}F”, the supernatant was removed by centrifugation at 10,000 rpm for 5 min and washed twice with DW.

^{68}Ga was eluted from a ^{68}Ge/^{68}Ga generator (Eckert&Ziegler, Obninsk, Germany) with 407 - 481 MBq activity per elution. After elution in 1 mL of 0.1M HCl, the $^{68}GaCl_3^+$ was

neutralized with 1 M NaOH solution, immediately added to HMSN suspension (500 µL of 0.1 M HEPES buffer) and incubated at 70 ˚C for 1 hour.

Reference

1. Holland, J. P., Sheh, Y. & Lewis, J. S. Standardized methods for the production of high specific-activity zirconium-89. *Nuclear medicine and biology* **36**, 729-739 (2009).

Jun Young Lee et al., Supplementary Fig. S1

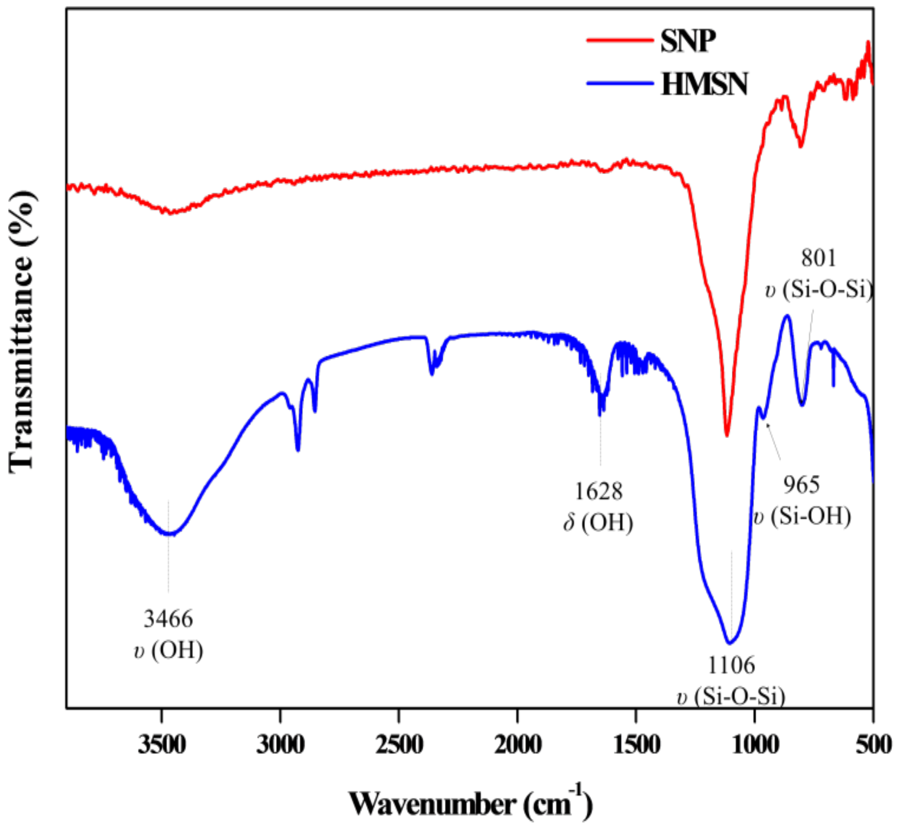

Supplementary Figure S1. FT-IR spectrum of prepared SNP and HMSN.

Jun Young Lee et al., Supplementary Fig. S2

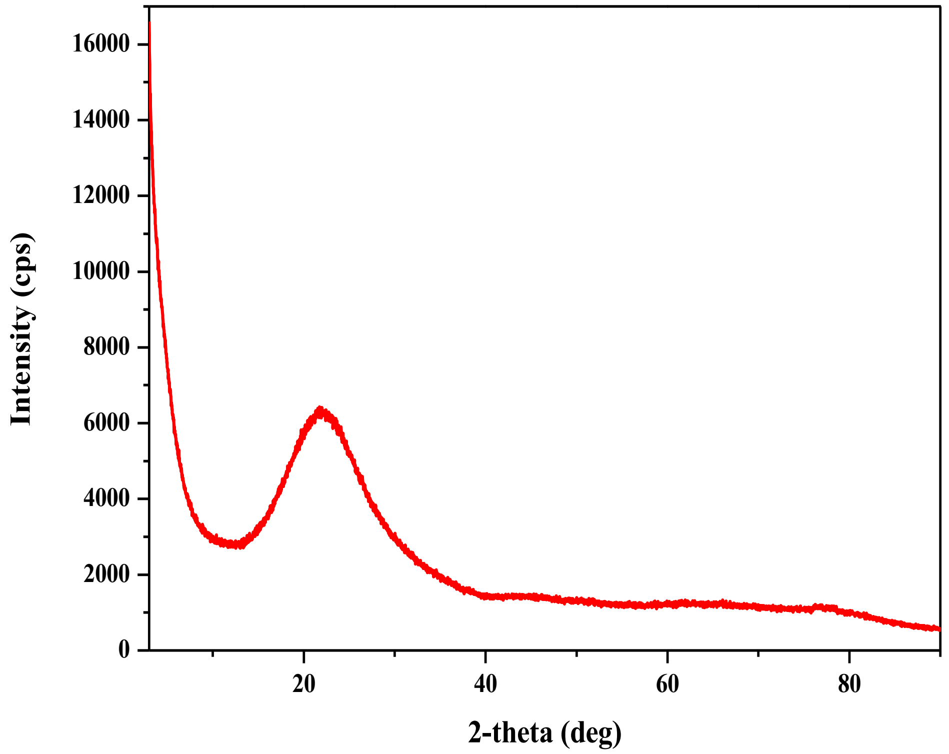

Supplementary Figure S2. Powder X-ray diffraction pattern of HMSN. A disordered mesostructure at 2-theta angle of 21°.

Jun Young Lee et al., Supplementary Fig. S3

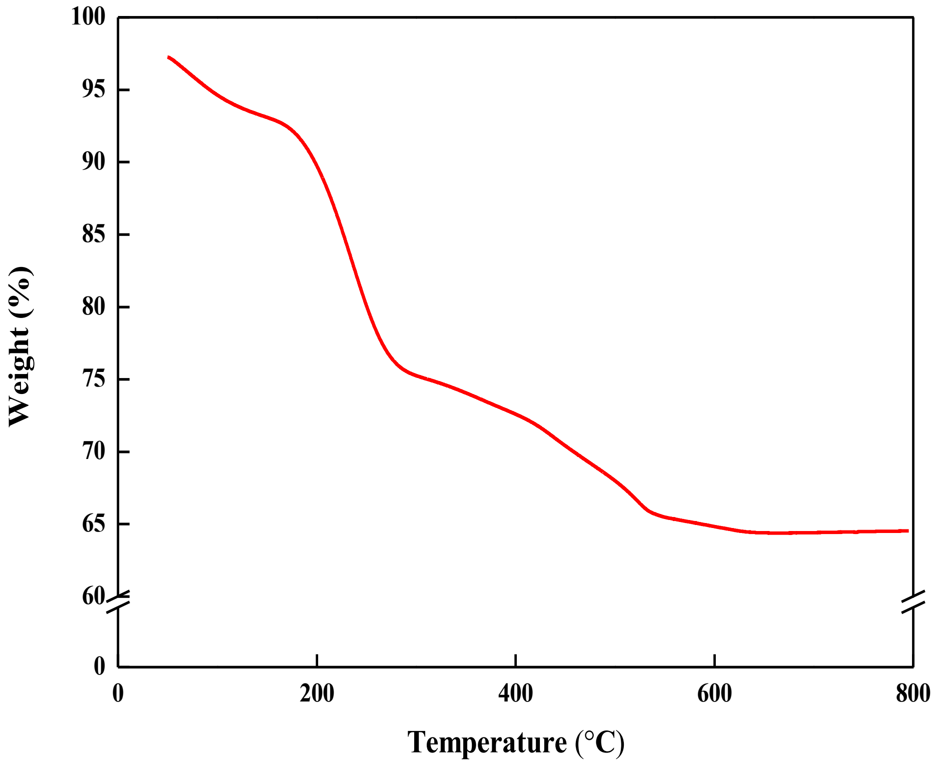

Supplementary Figure S3. TGA curve of HMSN in nitrogen atmosphere, heating rate: 10 °C/min.

Jun Young Lee et al., Supplementary Fig. S4

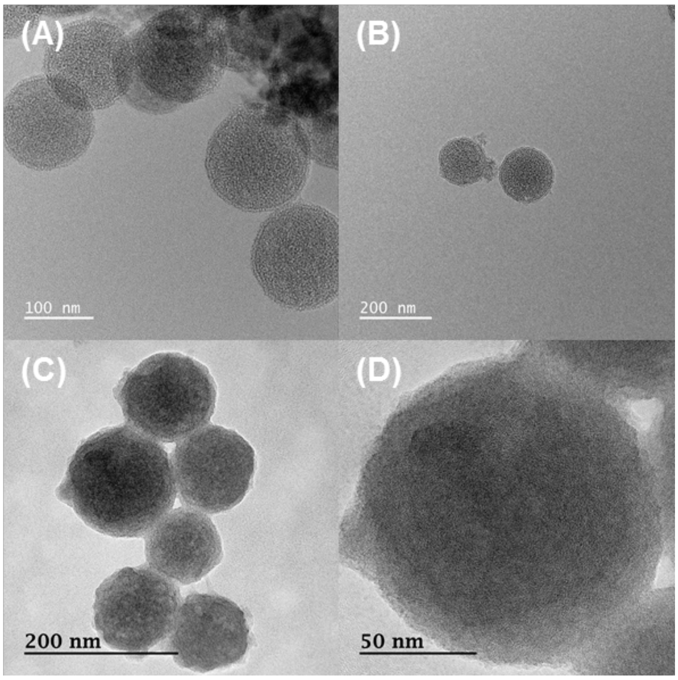

Supplementary Figure S4. Representative TEM images of (A, B) HMSNs and (C, D) Rm-HMSNs with well-dispersed biomimetic nanoparticles

Jun Young Lee et al., Supplementary Fig. S5

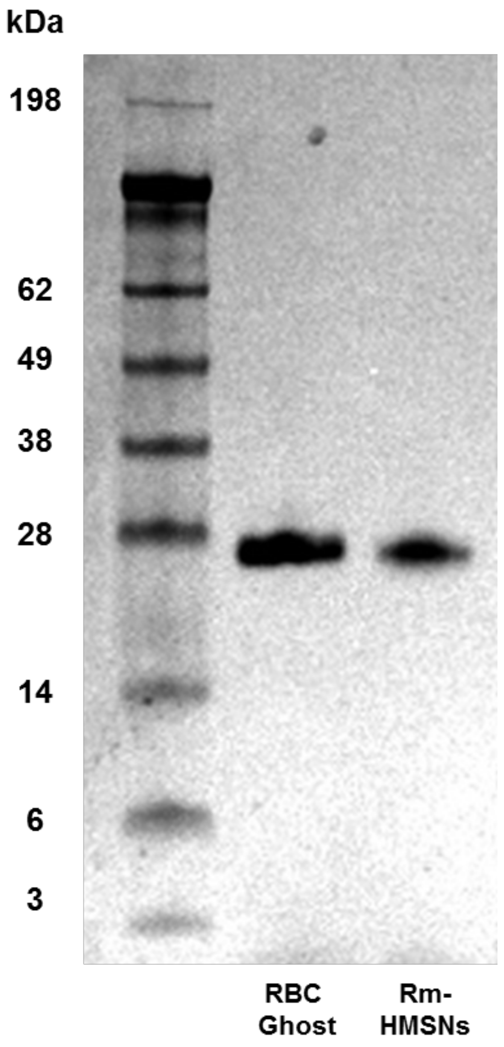

Supplementary Figure S5. Western blot results demonstrating that CD47 protein in intact with Rm-HMSNs

Jun Young Lee et al., Supplementary Fig. S6

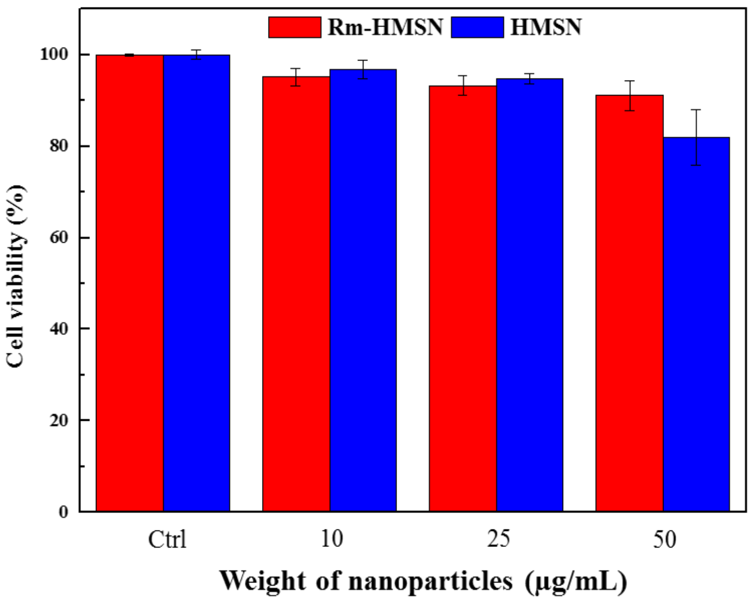

Supplementary Figure S6. Cytotoxic effect of Rm-HMSN and HMSN on the CT-26 cancer cell line measured by the MTT assay. Data are shown as mean ± SEM of three separate experiments.

SEM; standard error of mean

Jun Young Lee et al., Supplementary Fig. S7

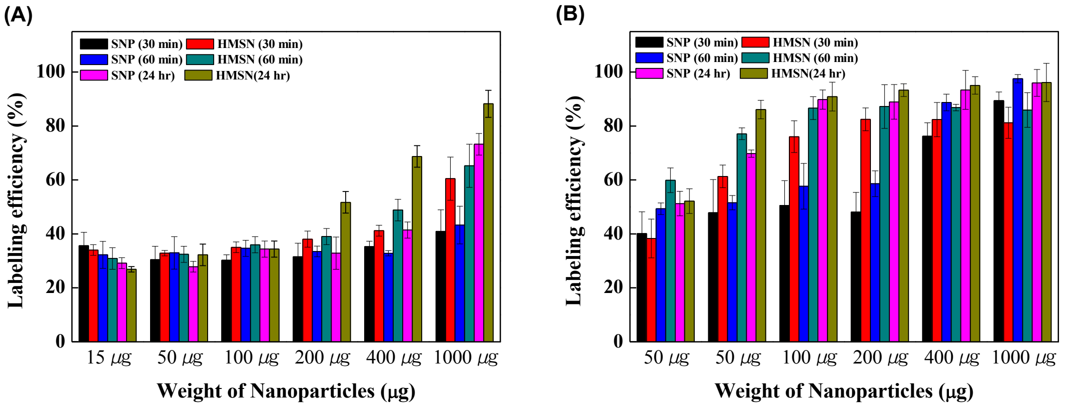

Supplementary Figure S7. Optimized labelling efficiency of chelator free ^{89}Zr-SNP and ^{89}Zr-HMSN using neutralized (A) ^{89}Zr-oxalate and (B) ^{89}Zr-chloride. Data are shown as mean ± SEM of three separate experiments.

Jun Young Lee et al., Supplementary Fig. S8

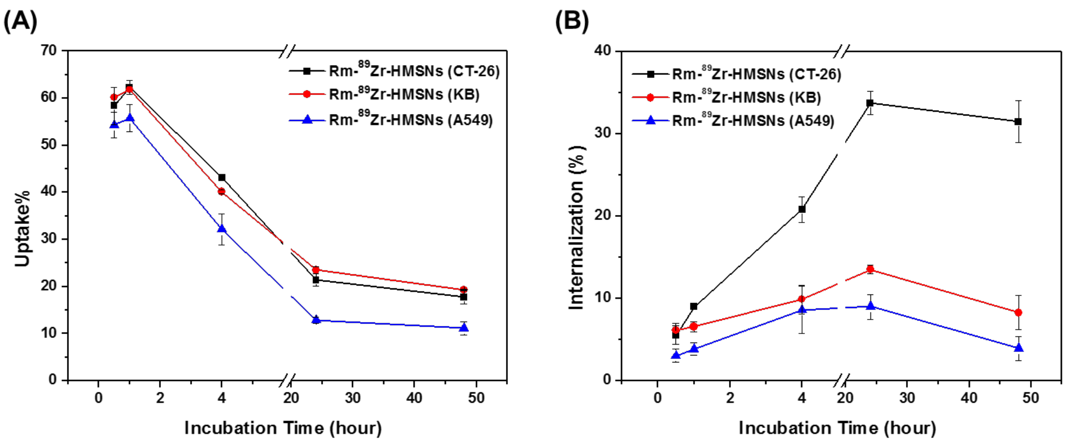

Supplementary Figure S8. Cellular uptake and Internalization of Rm-^{89}Zr-HMSNs into various cancer cell lines (CT-26, KB, and A549 cells) at a series of time points (0.5, 1, 4, 24, and 48 hour). Data are shown as mean ± SEM of three separate experiments.

Jun Young Lee et al., Supplementary Table. S1

	^{18}F-HMSN	^{68}Ga-HMSN
**Labelling efficiency**	2.93 ± 0.12	99.8 ± 1.64
**Stability in human serum (Incubation time: 30 min)**	28.7 ± 1.40	85.0 ± 1.22

Supplementary Table S1. Comparison of the labeling efficiency and stability of ^{18}F-HMSN and ^{68}Ga-HMSN as a positron emitter for PET tracer (n=3).

Jun Young Lee et al., Supplementary Table. S2

Incubation Time (hour)	^{89}Zr-HMSNs			Rm-^{89}Zr-HMSNs		
	Human serum	Cell culture media	0.9% NaCl	Human serum	Cell culture media	0.9% NaCl
**0.25**	91.57 ± 1.28	89.69 ± 1.75	91.61 ± 2.47	98.62 ± 0.67	98.64 ± 0.24	98.88 ± 1.08
**0.5**	86.41 ± 2.14	69.87 ± 1.21	92.31 ± 2.14	95.44 ± 0.34	92.44 ± 1.22	98.90 ± 0.94
**2**	89.84 ± 2.45	60.82 ± 1.33	92.00 ± 2.03	93.15 ± 0.47	89.65 ± 1.44	98.89 ± 0.12
**24**	57.22 ± 1.45	44.23 ± 2.04	89.28 ± 1.45	91.25 ± 1.54	85.39 ± 1.34	98.87 ± 0.34
**48**	43.62 ± 0.98	41.25 ± 1.48	88.12 ± 0.54	88.45 ± 0.54	81.24 ± 1.46	98.62 ± 1.34
**72**	42.31 ± 1.26	38.09 ± 0.87	86.37 ± 1.58	86.15 ± 1.66	76.98 ± 0.87	98.76 ± 0.87
**144**	26.62 ± 2.00	29.33 ± 1.62	80.97 ± 1.23	82.22 ± 1.24	75.55 ± 0.95	98.69 ± 0.94
**168**	18.33 ± 1.45	21.24 ± 1.34	79.22 ± 0.36	80.09 ± 1.63	67.03 ± 1.45	98.77 ± 0.62

Supplementary Table S2. Data were calculated from iTLC measurement and are presented as average ± maximum error (n=3).

Jun Young Lee et al., Supplementary Fig. S9

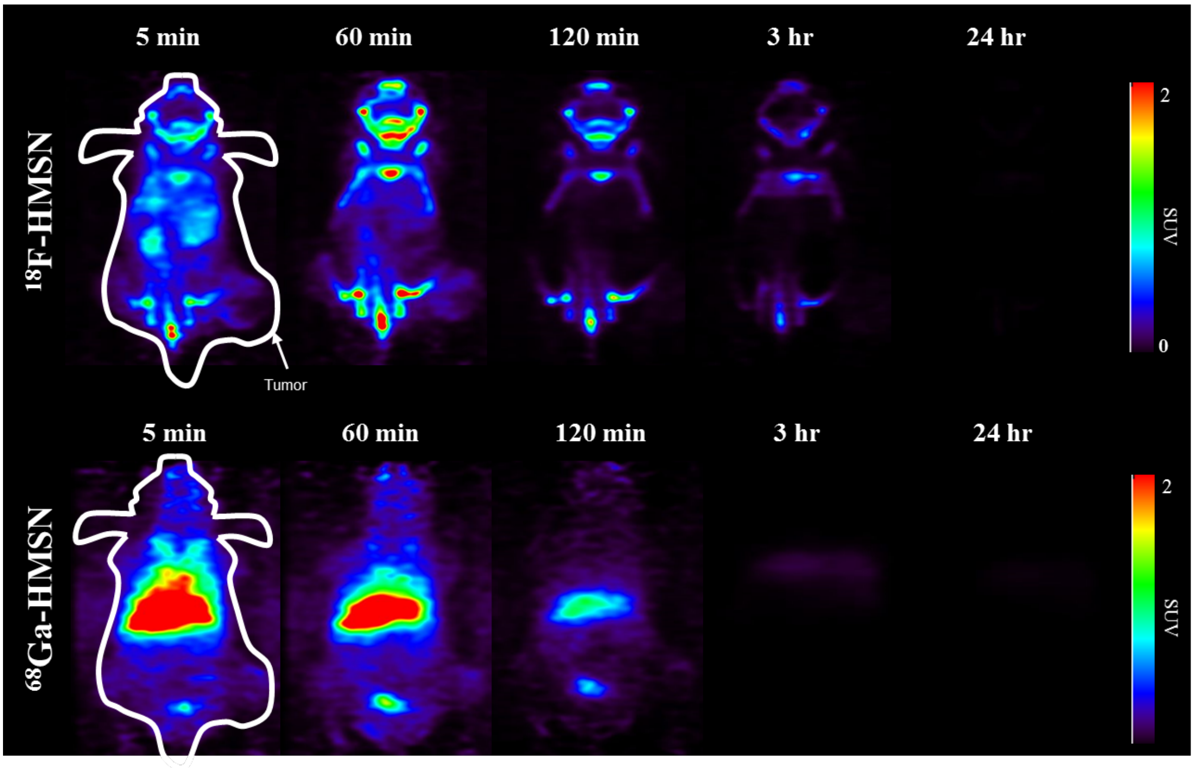

Supplementary Figure S9. *In vivo;* Small-animal PET images of ^{18}F-HMSN (top) and ^{68}Ga-HMSN (bottom).

Jun Young Lee et al., Supplementary Table. S3

	1 hr	24 hr	48 hr	72 hr	96 hr
**blood**	3.87 ± 0.26	7.11 ± 0.22	3.21 ± 0.15	3.85 ± 1.04	3.49 ± 0.34
**heart**	3.54 ± 0.53	4.18 ± 0.43	4.55 ± 0.11	4.34 ± 0.27	4.88 ± 0.69
**lung**	17.1 ± 1.61	5.93 ± 0.80	7.28 ± 1.42	5.31 ± 0.67	6.40 ± 0.50
**liver**	11.3 ± 0.93	14.2 ± 3.91	14.3 ± 1.24	16.3 ± 1.53	17.0 ± 1.58
**spleen**	13.8 ± 1.00	26.7 ± 4.93	29.8 ± 4.15	27.9 ± 7.01	41.5 ± 0.75
**stomach**	0.21 ± 0.04	0.23 ± 0.06	0.16 ± 0.01	0.14 ± 0.01	0.15 ± 0.01
**intestine**	0.09 ± 0.01	0.09 ± 0.01	0.08 ± 0.01	0.07 ± 0.03	0.05 ± 0.01
**pancreas**	0.40 ± 0.12	0.34 ± 0.16	0.31 ± 0.07	0.34 ± 0.13	0.56 ± 0.05
**kidney**	0.22 ± 0.02	0.19 ± 0.04	0.25 ± 0.03	0.18 ± 0.03	0.20 ± 0.01
**muscle**	0.12 ± 0.01	0.14 ± 0.01	0.25 ± 0.02	0.17 ± 0.04	0.22 ± 0.06
**fat**	1.27 ± 0.10	0.19 ± 0.03	0.52 ± 0.39	0.28 ± 0.13	0.20 ± 0.04
**bone**	1.21 ± 0.17	1.60 ± 0.40	3.14 ± 0.32	2.89 ± 0.61	3.41 ± 0.43
**skin**	0.28 ± 0.04	0.12 ± 0.01	0.11 ± 0.01	0.10 ± 0.04	0.06 ± 0.01
**tail**	2.37 ± 0.31	0.45 ± 0.02	0.55 ± 0.06	0.51 ± 0.22	0.50 ± 0.04
**tumor**	1.90 ± 0.07	3.47 ± 0.09	2.66 ± 0.15	2.13 ± 0.13	1.47 ± 0.17

Supplementary Table S3. Uptake value (% ID/g) (n = 3 per group) from a biodistribution study in which mice received Rm-^{89}Zr-HMSN 1 hr to 96 hr prior to tracer administration.

Jun Young Lee et al., Supplementary Table. S4

	1 hr	24 hr	48 hr	72 hr	96 hr
**blood**	0.49 ± 0.02	0.48 ± 0.01	0.83 ± 0.05	0.57 ± 0.08	0.42 ± 0.01
**heart**	0.54 ± 0.05	0.83 ± 0.04	0.58 ± 0.02	0.49 ± 0.03	0.30 ± 0.03
**lung**	0.11 ± 0.01	0.59 ± 0.03	0.37 ± 0.04	0.40 ± 0.04	0.23 ± 0.02
**liver**	0.16 ± 0.01	0.25 ± 0.04	0.18 ± 0.01	0.13 ± 0.01	0.08 ± 0.01
**spleen**	0.13 ± 0.01	0.13 ± 0.01	0.09 ± 0.01	0.07 ± 0.01	0.03 ± 0.01
**stomach**	9.22 ± 0.76	15.1 ± 1.94	15.9 ± 0.30	14.9 ± 0.97	9.56 ± 0.79
**intestine**	20.3 ± 1.20	36.0 ± 2.16	30.3 ± 1.30	35.0 ± 9.21	25.0 ± 0.85
**pancreas**	4.98 ± 0.81	11.8 ± 2.99	8.82 ± 1.36	7.25 ± 2.12	2.60 ± 0.21
**kidney**	8.44 ± 0.78	18.2 ± 2.03	10.5 ± 0.69	11.8 ± 1.77	7.22 ± 0.79
**muscle**	14.70.70	24.2 ± 1.79	10.7 ± 0.51	12.3 ± 1.47	6.88 ± 1.32
**fat**	1.50 ± 0.08	18.2 ± 1.56	7.10 ± 2.56	8.69 ± 2.19	7.53 ± 1.20
**bone**	1.58 ± 0.09	2.26 ± 0.34	0.85 ± 0.06	0.76 ± 0.10	0.43 ± 0.04
**skin**	6.84 ± 0.71	29.0 ± 1.23	22.5 ± 0.76	24.3 ± 6.38	21.7 ± 2.69
**tail**	0.81 ± 0.06	7.69 ± 0.11	4.79 ± 0.28	4.80 ± 1.21	2.95 ± 0.24
**tumor**	-	-	-	-	-

Supplementary Table S4. Tumor to blood and tissue ratios of mice (n=3 per group) from biodistribution results
